# Vaping Cessation Support Recommendations from Adolescents Who Vape: A Qualitative Study

**DOI:** 10.21203/rs.3.rs-4077848/v1

**Published:** 2024-03-20

**Authors:** Lori Pbert, Catherine E. Dubé, Catherine S. Nagawa, Dante P. Simone, Jessica Wijesundara, Rajani Sadasivam

**Affiliations:** UMass Chan Medical School; UMass Chan Medical School; Massachusetts General Hospital, Harvard Medical School; UMass Chan Medical School; UMass Chan Medical School; UMass Chan Medical School

**Keywords:** Vaping, e-cigarette use, adolescents, reasons to quit, cessation strategies, qualitative study

## Abstract

**Background:**

Youth vaping is an epidemic, being more prevalent than any other tobacco use. To inform cessation interventions, we explored what adolescents perceive to be their reasons for quitting and strategies to help in their quit efforts.

**Method:**

Semi-structured interviews were conducted with a convenience sample of 11 adolescents reporting vaping in the past 90 days and recruited from a high school in Massachusetts. Interviews were transcribed, and dual coded. Inductive thematic analysis was employed and thematic summaries were prepared.

**Results:**

Reasons adolescents reported for quitting included: cost; experiencing “nic-sick” from nicotine withdrawal or excess intake; negative impacts on mood, concentration, or health; and experiencing symptoms of nicotine dependence. Nearly all tried to quit multiple times. Barriers to quitting included: exposure to vaping; access to vape products; stress; and “cool” new products or flavors. Quit strategies included: avoiding others vaping; seeking social support to quit; addressing peer pressure to continue vaping; learning successful quit strategies from peers; and using distraction strategies or alternatives to vaping.

**Conclusion:**

Many adolescents who vape want to quit and most have tried multiple times. Interventions need to engage adolescents with varying reasons to quit, barriers, and quit strategy preferences.

**Clinical Trial Registration:**

This study is registered through ClinicalTrials.gov. The trial registration number is NCT05140915. The trial registration date is 11/18/2021.

## BACKGROUND

The FDA and the U.S. Surgeon General call the increasing e-cigarettes use among U.S. adolescents an epidemic. E-cigarettes use among this group has exceeded combustible cigarette use.^1^ The 2021 National Youth Tobacco Survey^2^ found that 14.1% of high school students reported current (past 30-day) e-cigarette use, with more than a quarter (27.6%) using e-cigarettes daily and almost half (42.3%) using them on 20 or more of the past 30 days. Although e-cigarettes use may have declined during height of COVID-19 pandemic, recent estimates suggest that the rates are again increasing.^3^

Peer influence as well as appealing flavors have been identified as major factors in adolescent e-cigarette use. Adolescents do not recognize that the most popular e-cigarettes contain nicotine,^4^ risking harm to brain development, impacting learning, memory, attention, mood, and impulse control, and increasing the risk of addiction.^5^ Adolescents perceive e-cigarette use to be low risk, leading to a low intention to quit.^6^ Effective cessation interventions are lacking, with only one that exists (This is Quitting texting program from the Truth Initiative) to our knowledge.^7 8 9^ While This is Quitting^9^ generated a high demand for cessation support and encouraging evidence of success, a variety of cessation tools are needed especially for less motivated adolescents, including tools that focus on the pervasive influence of peers on e-cigarette use.^6,10^

This formative research was part of a pilot study developing peer-driven text messaging and coaching mobile intervention to support cessation for adolescents who vape. We conducted in-depth qualitative interviews to explore adolescents’ reasons to quit vaping, prior attempts to quit, and their recommendations for cessation strategies.

## METHODS

We detailed methods for this research in a previously published companion manuscript.^11^

### Interview Guide Development

A qualitative, open-ended, semi-structured interview guide was developed. Areas of inquiry included reasons to quit, advice to friends thinking of quitting, prior quit experiences, and recommended strategies to help quitting (Appendix 1).

### Setting and Participant Recruitment

A convenience sample was recruited from one public high school in Massachusetts from among 513 students. High school enrollment consisted of 82% white, 10% Hispanic, 5% multi-race non-Hispanic, 2% African American, and 1% Asian, with median household income of approximately $80,000. Flyers posted in the school, school-wide announcements, and materials mailed to parents were used. Parents could opt their child out if desired. Potentially eligible participants who reported having vaped nicotine in the last 90 days were identified and screened. Assent (for those under 18 not opted out by their parents) and consent (for participants over 18 years old) procedures were conducted prior to enrollment and data collection. Participants were compensated with a $20 gift card.

### Data Collection

Semi-structured open-ended interviews were scheduled for 1 hour during the school day in a private room and recorded. A HIPAA trained transcriptionist transcribed recordings. All transcripts were reviewed and verified by a research team member.

### Analytic Approach

Inductive thematic analysis was employed using a coding start-list based on protocol questions (deductive coding) with additional coding driven by immersion in the qualitative data (inductive coding).^12^ Using Dedoose qualitative analysis software,^13^ two of the authors) coded all interviews. Disputes in coding decisions were discussed until consensus was reached. Coding tests for inter-rater reliability^14^ resulted in a Kappa >.94. For each code (Table 1), reports including all coded quotations were generated and code summaries were then prepared by two team members, identifying areas of agreement and the range of responses for each code. Consensus code summaries formed the basis for results reported below.

## RESULTS

### Participants

Thirty-two students were screened; 12 were eligible and enrolled. One participant dropped out resulting in a final sample of 11. Three identified as female, 4 as male, 3 as non-binary, and 1 declined. The majority were 10th or 11th graders (n = 8), non-Hispanic White (n = 8), low income as defined by participation in the free lunch program (n = 7), had never smoked traditional cigarettes (n = 7), reported being addicted to vaping (n = 7), used flavors (n = 10), felt moderately to extremely confident in their ability to quit (n = 9), and smoked marijuana in the past month (n = 8). All vaped nicotine (n = 11). Half reported not thinking of quitting (n = 5), the others reported already being quit (n = 4) or considering quitting (n = 2).

### Qualitative Results

Eleven individual interviews were conducted between March and June 2022 (average = 32.5 minutes; range 26–43 minutes). Five major themes were identified related to adolescent vaping cessation (Table 2 includes illustrative quotes).

### 1. Reasons to quit vaping

#### 1.1 Cost

Many participants described vaping as expensive, pricey, a “waste of money” and “not worth it.” 1.2 “Nic-sick”Several participants experienced “nic-sick”, unpleasant physical symptoms (nausea, headaches) attributed to nicotine withdrawal or excessive nicotine intake from vaping. 1.3 Physical health concerns about the effects of vaping on their physical health included cough, reduced lung capacity/shortness of breath, asthma, sore/burned throat, as well as concerns about future health including cancer risk.

#### 1.4 Negative effect on mood and concentration

Participants mentioned irritability, frustration, “depressive mood swings,” worsening anxiety, and distraction, and inability to concentrate.

#### 1.5 Addiction

Addiction was seen as a main reason to quit for many participants. While some used the term “addiction”, many reported symptoms of nicotine dependence without associating their experience with dependence. It was described as an unwelcome feeling such as significant cravings to vape when they are unable to vape, see others vape, when stressed, and when they realize they do not have access to vaping products. When unable to vape, other symptoms included feeling desperate, anxious, depressed, impatient, restless, unable to concentrate, irritable, and headaches. A few participants reported never experiencing cravings to vape but recognized it in their friends.

#### 1.6 Family and friend disapproval

Only a few participants discussed family disapproval as a significant negative aspect of vaping. One mentioned friend disapproval and another felt being viewed as a vaper by others was a negative and didn’t want to be seen that way.

### 2. Prior experience with quitting vaping

#### 2.1 Ever tried to vape less often or stop vaping and if so, number of times tried

All participants except two reported having tried many times (2–4 or more times) to vape less often or stop vaping but gave up because they “need it”. Only one participant reported successfully quitting after one try.

#### 2.2 What cutting back/stopping was like for them

Of participants who had tried to quit or cut back, the majority noted that it was very difficult, experiencing irritability and stress.

#### 2.3 Why decided to cut back/stop

Several participants reported deciding to quit because they were concerned about their future health. One noted trying to quit due to cost and another was afraid of being suspended from school.

#### 2.4 What helped quit

What helped in quitting varied greatly across participants. Having fun with family, being with friends, willpower, reducing stress, avoiding others who were vaping, talking about vaping, or using marijuana were seen as helpful strategies.

#### 2.5 What made quitting harder and reasons for relapsing

Challenges to quitting included being exposed to others vaping, stress from family or school, and learning about new e-cigarette flavors. Not surprisingly, many of these challenges were also cited as reasons for resuming e-cigarettes the last time they tried to stop, including stress, family stress, seeing a new “cool” vaping product, and access to vape products through friends. One did not know why they resumed vaping.

### 3. Confidence in their ability to quit

#### 3.1 What would help them become more confident in their ability to quit

Participants mentioned that an optimistic attitude, achieving goals, avoiding those who vape, and quitting with their friends would increase their confidence in quitting. A few participants reported that they already had sufficient confidence in their ability to quit but were not interested in quitting.

### 4. Strategies to quit

#### 4.1 Avoidance of others vaping and addressing peer pressure to not quit

Avoiding those who are vaping and places where vaping occurs, along with not asking friends for a vape, were key strategies. The importance of peer pressure was noted as a significant barrier to successfully quitting vaping.

#### 4.2 Soliciting social support

Several participants reported getting support from their social network, including friends or family, to help them quit. Having others quitting with them was considered particularly helpful.

#### 4.3 Distraction strategies and alternatives to vaping

Finding ways to distract from cravings and finding things to do instead of vaping were also mentioned, like playing games, chewing gum, eating a mint, going for a ride, and doing something of interest.

#### 4.4 Peer role models who had successfully quit

A few participants noted learning of quit strategies from peers who had successfully quit. They mentioned not knowing much about the process of quitting and related challenges and barriers, so having others share their quitting journey was important.

#### 4.5 Use nicotine replacement therapy (NRT) or cannabis

Many participants had adults in their lives who had used NRT to quit smoking cigarettes. They reported mistakenly assuming that NRT could be used by adolescents in quitting vaping (NRT is not FDA approved for those under 18 years of age in the USA). A few suggested using cannabis to help with quitting, reporting a similar calming effect to vaping.

### 5. Technology-based interventions recommended

Most participants endorsed text messaging. Other suggestions included social media, email, pop-up ads, a timer that regulates vaping intake, a placebo vaping device that is only water, and an unrelated video game intended as a distraction

## DISCUSSION

Most of the adolescents interviewed reported wanting to quit vaping and having tried unsuccessfully to quit many times. This finding is consistent with prior findings of high interest in quitting e-cigarette use,^15^ suggesting an unmet need for vaping cessation interventions.

Common reasons to quit vaping reported included cost, cravings, symptoms of nicotine addiction (getting “nic-sick” from excess nicotine intake or withdrawal), and significant concerns regarding the effect of vaping on their health and their mood including worsening of anxiety, depressive symptoms, stress, and their ability to concentrate. Of note, many reported using vaping to try to reduce their symptoms of anxiety and stress. Anxiety disorders are the most common psychiatric condition in youth, affecting nearly 1 in every 4 adolescents.^16^ Given nicotine is known to produce an anxiolytic effect,^17^ it makes sense that adolescents would try vaping nicotine to manage their anxiety. Unfortunately, these adolescents also reported that vaping made their anxiety worse, not better, due to withdrawal symptoms experienced between vaping sessions.

Adolescents also noted many barriers to their being able to quit. One key barrier was that their friends vape, resulting in their being exposed to vaping and vaping products on a regular basis. Peer use has been noted as key reason for initiation as well as the continuation of adolescent vaping. Other barriers included vaping for mood management and the appeal of new vaping products and flavors.

Adolescents shared many potential strategies to quit including avoiding exposure to vaping, seeking support, learning from peer quitters, resisting peer pressure to vape, and various distraction strategies. Two strategies mentioned by the participants are not appropriate for adolescents including nicotine replacement therapy (which is not FDA-approved for use in youth under the age of 18 and has not yet been found to be effective in helping youth quit), and the use of cannabis which is illegal in most states and all US states under the age of 21. Some adolescents did discuss dual use of e-cigarettes and cannabis, which needs to be systematically evaluated, as cannabis use potentially increases quitting di culty and lead to more serious psychoactive drug use.^18^

There were limitations to our study. First, due to challenges faced by schools during the COVID-19 pandemic, recruitment was difficult. However, we were able to recruit 11 participants and reached saturation on many of the constructs assessed, including reasons for quitting and strategies to quit. Second, there were few participants who had successfully quit vaping and maintained abstinence. Expanding the sample to include both successful quitters and those who struggle to quit in future studies will provide rich data on both challenges and effective strategies used by this population.

## Figures and Tables

**Table 1:** Codes Included in the Analysis

Code Name	Definition
Reasons2Quit	Reasons participant or others should quit vaping. Includes: Benefits of quitting, what dislike about vaping, concerns about vaping (e.g., impact on important things in life like sports, ability to concentrate on schoolwork, financial costs, impact on mood, health, friendships, effect of nicotine)
Advice	Advice participant would give to a friend thinking of stopping vaping
PriorQuitAttempts	Reasons for deciding to try to cut back/stop, how often made attempts, what specific strategies were used, use of other tobacco product/vaping product to cut back/quit, strategies that helped/worked well when trying to cut back/stop vaping, supportive/helpful others, what made it hard to stop vaping (e.g., friends or family who interfered with efforts, mood/anxiety or stress), what made participant go back to using e-cigarettes last time tried to stop/vape less
QuitConfidence	What would help them become more confident in their ability to quit
QuitStrategies	If participant decided to quit, how would they go about it, what strategies do they think would help them stop
Technology-basedInterventions	Technologies that might help participant and friends quit vaping

**Table 2:** Selected Theme and Representative Quotes

Themes	Subthemes	Illustrative Quotes
1 Reasons to quit	1.1 Cost	ID02 *It’s an expensive piece of electronics*.
		ID10 *It’s a big expense. You’re gonna do that and get addicted, then you’re going to be fresh outta cash*.
		ID11 *It’s expensive… It’s really not worth it. You save a lotta money not doing it, save yourself a lotta trouble…*
	1.2 “Nic-sick”	ID04 *Sometimes it makes me feel a little sick, so that can sometimes affect certain things like just hanging out with friends. They call it nic-sick. If I feel nic-sick … I’ll kinda go quiet, ‘cause I’m trying not to throw up, ‘cause it makes you a little nauseous… I was chiefmg… taking hit after hit..and I think I accidentally maybe took a couple too many hits, ‘cause I was in the car with my dad, and I started to feel sick. And I had to roll down the window and throw up*.
		ID11 *Sometimes it’ll give you random headaches. Your stomach will randomly hurt. And obviously if you haven’t had it for a while, that doesn’t feel good*.
	1.3 Effects on physical health, including cancer risk	ID02 *Health concerns, lung cancer. You start getting really bad coughs. You might develop asthma due to the smoke… I realized that it was going to cause me future health problems*.
		ID04 *I’ve heard it kind of makes your lung capacity… a little smaller. People say they have a harder time catching their breath*.
		*ID03 How bad it is for you… I wanted to go to the gym more, and I wanna be able to run longer distances without being outta breath*.
	1.4 Negative effect on mood and ability to focus	ID07 *I thought that vaping was helping my anxiety, but it really just made it worse… Sometimes it makes me more stressed … it’s like during the day I’ll just be thinking, “Oh, I wanna go home. I wanna go hit it,” and it would just completely get me off-track from my schoolwork*.
		ID04 [When unable to vape] *I feel like it makes it harder to focus on things for sure. I feel like I become a little bit more irritable. People can get on my nerves a lot easier if I hadn’t had any nicotine* … *It can definitely make me feel annoyed, not so much angry as maybe frustrated… Anxiety a little bit*.
		ID06 *I feel like it gets in the way with school, and I feel like that’s a big problem with me*.
		ID01 *People get depressive mood swings and stuff like that, because they don’t get nicotine*.
	1.5 Addiction	ID06 *I just thought it would be cool, and then I didn’t realize that it would get me addicted to it. [I dislike] how I feel like I need it. I feel desperate, in a way. I need it …and then when I actually do it, then I’m like, is it really worth it?*
		ID06 *I feel like my cravings have been just escalating, and I feel like when I get upset, I’m like, “Oh, my God, I need this.” I feel like I need it, in a way, and I feel like nicotine will help me, which - I know that it won’t, but in my head, I just think it will help me*.
		ID12 *I just kinda think about it a lot, and then once I finally do vape, I feel better. So [that’s] what addiction is*.
		ID10 *Whenever we’re hanging out and there’s not vapes around, some of our friends will be like, “Oh, I wish we had it. I need it.”*
	1.6 Family and friend disapproval	ID10 *They [family] don’t know, but if they ever found out, I would be chalked up on the side of the road*.
		ID09 *I don’t like that I vape most of the time… I don’t want to be seen like that at school… I prefer to be seen in a more professional way, and I feel like that isn’t vaping*.
		ID07 *The stress and the anxiety and the fear that your parents found it*
		ID10 *Specific people won’t respect you because of what you do… I’ve lost a couple of ‘em [friends]*.
2 Prior experience with quitting vaping	2.1 Ever tried to vape less often or stop vaping and if so, number of times tried	ID06 *I’ve tried many many times, but I just give up, and I feel like that I just need it*.
		ID12 *Two or three times….unsuccessfully*
		ID11 *…failed four times*.
		ID10 *No… Because I don’t really do it that often as it stands. And quitting altogether, I could do that. I just don’t really feel or see the need to, really…*
	2.2 What cutting back/stopping was like for them	ID01 *It was kinda hard, ‘cause you have it around you all the time, people vaping around you*.
		ID11 *It’s terrible*.
		ID12 *It was hard. I tried to only hit it three times at most three sessions, I guess, a day, and I found myself being really irritable and stressed and couldn’t really handle people*.
		ID05 *It was pretty smooth. I smashed the device I had, and I was like, “I’m done.” And then I was done. That’s when I quit. I was just like, “All right, I’m done.”*
	2.3 Why decided to cut back/stop	ID02 *Because I realized that it was going to cause me future health problems…*.
		ID11 *‘Cause I got suspended for the first time, and I got scared*.
		ID01 *You know the health issues. Also, you just don’t feel like yourself sometimes when you’re vaping. It feels like that’s not you*.
		ID05 *Because I saw a video that a guy got put in the hospital, and I was like, “I don’t want that to be me.”*
	2.4 What helped quit	ID01 *Honestly, friends telling me that they’re trying to quit, too, motivational*.
		ID06 *Probably when I’m with my family doing things as a family. We’re not thinking about any drugs or - we’re just having fun*.
		ID02 *Support from my friends, definitely, but mostly… willpower to not wanna be like my mother. I have a goal I need to upkeep*.
		ID12 *Trying to cut out other stressors in your life, ‘cause stress is a big factor of why I started*.
		ID05 *Friends, video games and candy helped*.
	2.5 What made quitting harder and reasons for relapsing	ID01 *It was kinda hard, ‘cause you have it around you all the time, people vaping around you*.
		ID03 *Having all my friends do it*.
		ID05 *Everyone asking me if I wanted a hit*.
		ID07 *Every now and then I would…notice,…”Oh, this one looks fancy. I wanna try this one,” or, “Oh, this sounds like a really good flavor. I wanna try that.” So, it’s like a bunch of stuff came up to the point where it’s like, I just couldn’t quit. So, then I bought one, and I was like, OK, this is my last one. After this, I’m done. And then I opened social media, and I found this one that looked really cool. I’m like, OK, this is gonna be my last one. And it was not*.
		ID12 *My family. They’re my main reason why I started. They’re very stressful, and once I started vaping…I could handle my family*. [I relapsed because] *I was really stressed out…and I was like, oh, OK. Never mind. I’m going back to this. I think it was actually a family thing, and my whole family was over, and* I *was all stressed out and stuff*.
3 Confidence in Quitting	3.1 What would help them become more confident in their ability to quit	ID12 *Seeing my friends do it [quitting] or having a group doing it with me, ‘cause if my friends are all doing it and I’m the only one that’s not, it’s like, damn*.
		ID09 *Not being around it*.
		ID03 *Probably stay away from people that do it*.
		ID11 *Nothing, really. I’m confident. I just don’t want to*.
		ID10 *I am so confident in my ability to quit. I could do it if I wanted to. I just don’t want to because I really just don’t care*.
4 Strategies to Quit	4.1 Avoidance of others vaping and addressing peer pressure to not quit	ID01 *Try to stop staying around it*.
		ID06 *Probably not going to the bathroom at all, ‘cause that’s where everyone does it*.
		ID06 *Some friends will try to push it like, “Are you sure? Are you sure?” and… if I’m really focused on trying to quit, I’ll be like, “No. I said no.”*
		ID05 *Pull yourself away from people that try and give you it*.
		ID12 *Throw away all my everythings of it and tell my friends that I’m going to quit and not to do it around me or talk about it*.
	4.2 Soliciting social support	ID11 *Stick to your friends for support and your family, too, ‘cause they’re the ones that - if you have friends that don’t do it, they’ll be very supportive of you stopping*.
		ID03 *Yeah, talking with friends definitely, maybe a family member or…someone you’re close to*.
		ID12 *Having other people around you that’s going through the same thing is cool and also having one-on-one time if you need it*.
	4.3 Distraction strategies and alternatives to vaping	ID06 I *try to not think about it. So,* *I try to find things to distract me like cleaning my room or doing homework*.
		ID05 *Just distract yourself a lot*.
		ID01 *Try to find other means of stress relievers instead of vaping*.
		ID02 *I would wean myself off of it, and I would start chewing gum or going for a walk*.
		ID12 *Chew gum. Do other things, hobbies that make you happy*.
		ID09 *Stupid games or talking with friends, doing something that interests you*.
	4.4 Peer role models who had successfully quit	ID06 *I’ve had many friends that completely stopped and made it successful for them… the successful ones…they just said no to the people like, “No, I’m not doing that anymore.”*
	4.5 Use NRT or cannabis	ID12 *Chew gum. Nicotine patches if they can get them*.
		ID10 *My aunt uses nicotine gum. She has used it for a while. My friends probably don’t even know that it exists*.
		ID11 *[CBD]gives a similar calming effect without having the negative side-effects of breathing in things*.
		ID09 *I’ll probably look for other things to do like play games or draw or eat something or smoke weed*.
5 Technology-based Interventions	5.1 Technologies to help with quitting vaping	ID04 *Maybe a water-vapor thing, ‘cause a lot of it is just the act of puffing on something*.
		ID01 *Any online things on social media*.
		ID09 *I probably would prefer things that didn’t have anything to do with it [vaping]. So, if we’re playing the video game, it would be nothing about vaping. … you would give scenarios for if you were doing the game, you could choose random scenarios that were semi-lighthearted or whatever*.
		ID05 *E-mail, because everyone’s refreshing their e-mails a lot. Yeah, that’s probably about it, text messages, videos*.

## Data Availability

The data underlying this article cannot be shared publicly due to the concerns for the privacy of individuals that participated in the study. Deidentified data will be shared on reasonable request to the corresponding author and approval to the UMass Chan Medical School IRB. Proposals should be submitted to Rajani.Sadasivam@umassmed.edu
